# Effect of incorporation of snail meat powder on sensory attributes and consumer acceptability of sorghum‐wheat buns

**DOI:** 10.1002/fsn3.2798

**Published:** 2022-03-01

**Authors:** Fredrick B. Agengo, Arnold N. Onyango, Charlotte A. Serrem, Judith K. Okoth

**Affiliations:** ^1^ 118985 Department of Human Nutrition Sciences School of Food and Nutritional Sciences Jomo Kenyatta University of Agriculture and Technology Nairobi Kenya; ^2^ 272337 Department of Consumer Sciences School of Agriculture and Biotechnology University of Eldoret Eldoret Kenya

**Keywords:** buns, compensation, consumer acceptability, low‐lysine, protein, sensory attributes

## Abstract

Formulation of foods from –low‐lysine cereals fortified with animal protein is a potentially sustainable approach to enhance protein quality in diet due to nutritional compensation while buns are valuable vehicles to deliver nutrients to human body because of their relatively noble eating quality and extended shelf life. The aim of this study was to evaluate the sensory attributes and consumer acceptability of sorghum‐wheat buns containing snail meat powder (SMP). Buns were prepared by replacing 5, 10, 15, 20, and 25% of sorghum‐wheat composite flour with SMP. Principal component analysis (PCA) revealed 99% total variation of 23 attributes for buns scored by a descriptive sensory panel, of which 98% was due to the proportion of SMP that replaced sorghum‐wheat composite flour in buns and the remainder 1% was due to the buns’ physical appearance. Compositing sorghum‐wheat buns with SMP imparted positive consumer attributes of fine crumb, sponginess, and crumby texture. It also resulted in buns with reasonably high sensory acceptability as evaluated by 8‐ to 9‐year‐old school children. The buns can serve as supplementary rich sources of protein for alleviating the menace of protein energy malnutrition in sub‐Saharan Africa.

## Graphical abstract

Compositing sorghum‐wheat buns with snail meat powder imparts positive consumer attributes of fine crumb, spongy, and crumby texture.

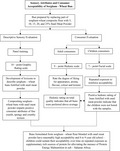

## 1 INTRODUCTION

Currently, sub‐Saharan Africa is experiencing a tremendous population surge brought about by growth and development of small urban settlements into big towns and cities (Okpala, 2009). This has led to “nutrition transition” as consumer demand for staple foods has declined with an increase in desire for more convenient products such as bakery products as important sources of nutrients in diet (Saranraj & Geetha, 2012). Wheat flour is the main ingredient in baking because of its unique qualities of holding and retaining water vapor and carbon dioxide resulting in spongy texture of baked foods (Odedeji et al., 2014). However, the bakery industry is at present faced with the challenge of increased wheat prices because of reduced agricultural land due to urbanization (Polat et al., 2016) and unfavorable climatic conditions in the tropics (Opara et al., 2013). This has generated interest in the utilization of alternative cereal flours such as sorghum in formulation of baked products (Salim et al., 2017).

Sorghum is an important drought‐resistant crop that thrives well in marginal lands where other cereals have failed (Muui et al., 2013) and is a principal source of protein and energy to millions of resource‐underprivileged households in the developing countries (Elkhier & Hamid, 2008). Primarily, its grains are ground into flour and consumed in the form of thin or stiff porridge (Anyango et al., 2011), but may also be incorporated in wheat flour at varying proportions to enhance dough volume, gas retention, viscoelastic properties, and crumb uniformity of baked foods (Esteller et al., 2005). Conversely, sorghum nutritional quality is low owing to its major storage protein kafirin that is poorer in indispensable amino acid lysine (Serrem et al., 2011) and has low digestibility on wet cooking (Duodu et al., 2003). Therefore, blending with highly digestible protein foods could enhance the nutritional value (Serrem et al., 2011).

Formulation of baked products from low‐lysine cereals incorporating snail meat powder (SMP) has been proposed as one of the most sustainable approaches to enhance protein nutritional quality in diet (Agengo et al., 2020a). Snail meat is an excellent source of protein that equals other conventional animals in indispensable amino acid lysine (Ebenebe, 2000) and is an important source of minerals and vitamins (Engmann et al., 2013). Notwithstanding, snail meat is underutilized in the diets of most Kenyans and in the African continent due to cultural repugnance, lack of familiarity, and poor attitude toward its consumption (Meyer‐Rochow, 2009). Therefore, consumption of snails may be enhanced through their integration into modern human diet through fortification. Blending cereals with snail meat results in nutritional compensation (Agengo et al., 2020b). Buns are valuable fortification vehicles due to their relatively noble eating quality and shelf life potential (Sharma et al., 2013).

New food products developed for children must be evaluated by children themselves exploiting simple but more reliable methods to measure preference (Guinard, 2001) on repeated exposure to reinforce acceptability (Wiejzen et al., 2008). Facial scales are more effective with children (Latorres et al., 2016) as they employ images that stimulate attention in respect to sensory acuity (Popper & Kroll, 2005). Leon et al. (1999) demonstrated food acceptability with 4‐ to 10‐year‐old children and established that 8‐ to 9‐year‐olds were more consistent in describing their liking. No previous studies have developed lexicon to describe buns incorporated with SMP using a trained panel. Likewise, no studies have employed 8‐ to 9‐year‐old on repeated exposure to determine long‐term acceptability of buns incorporated with SMP. This study evaluated the effect of incorporating with SMP on sensory attributes and consumer acceptability of sorghum‐wheat buns.

## 2 MATERIALS AND METHODS

### 2.1 Experimental design

Descriptive sensory and consumer acceptability studies with children followed a randomized complete block design (RCBD) to evaluate the six formulation of buns as treatments, which were randomized using three digit blinding codes and replicated thrice. The panelists were the units of assessment and evaluation sessions were the blocks. The adult consumer study followed a completely randomized design (CRD) approach. Randomized three‐digit code was assigned to each bun for blinding purposes with sample arrangement on trays being randomized for each panelist. The evaluation process was also randomized with evaluators coming to the evaluation room at random to evaluate samples for acceptability.

### 2.2 Formulation and preparation of buns

Buns evaluated in this study were prepared as described by Agengo et al. (2020a). Sorghum‐wheat composite flour was formulated in line with acceptable cereal blend at a ratio of 7:3 (Ayo & Nkama, 2003). Flour for buns was prepared by replacing part of composite flour with 5, 10, 15, 20, and 25% SMP. Six formulations of buns were developed according to the procedures of Pyler (1988) and those of 0% SMP served as control. Buns were allowed to cool to room temperature, after which they were weighed and packed in separate zip lock plastic bags.

### 2.3 Sensory evaluation

#### 2.3.1 Descriptive sensory evaluation

A total of eight trained panelists from both gender were recruited through an advert to participate in a descriptive study of sorghum‐wheat bun fortified with SMP. The panel was selected following the principle proposed by Plemmons and Resurreccion (1998) to have committed individuals aged between 18 and 64 years who were nonsmokers and did not suffer any food allergies to participate in the study. Those that responded attended an orientation session and were subjected to three different screening tests to determine their sensory acuity. The tests included identification of basic sweet, sour, bitter, salt, and umami tastes as described by Lawless and Heymann (2010), aroma identification, and a test to identify differences in sensory attributes that described taste, aroma, flavor, and appearance of buns. Before the tasting exercise, the panelists were asked to fill in a consent form that informed them of the nature of samples they were to evaluate. The panelists were trained in 12 sessions, each lasting about 2 h per day, over a period of 3 weeks. The generic descriptive method described by Einstein (1991) was used to execute the descriptive sensory profiling of buns. During the trainings, the panelists described that the differences that existed between samples and food items were used as references to clarify the sensory attributes. Panelist agreement was evaluated through a series of tests during the training. The panelists generated descriptors that were grouped under appearance, aroma/smell, flavor, texture, and after taste, with their definitions and reference standards to anchor the scale ends. Evaluation of buns was carried out over a period of 3 days in three sessions lasting about 45 min each a day. During each session, all the six formulations of buns were randomly presented to each panelist as a ¼ bun in a glass ramekin covered with cling film on a white tray, accompanied with a toothpick, serviette, carrot slices, and a plastic tumbler filled with distilled water for cleansing the palate in between tasting of the samples. Each sample was labeled with a random three‐digit code and the order of sample presentation was randomized. In addition, each panelist received a written methodology of assessment and a list of descriptors with their definitions. Reference samples were available to the panelists throughout the evaluation. The evaluation session was conducted in a food laboratory with each panelist seated at individual station where they were not able to see each other. The 23 descriptors (Table 1), developed by the panelists during their training, were used to rate the six samples on a 10‐point graphic rating scale to measure the intensity of individual attributes. Results were entered manually into the ballot.

**TABLE 1:** Descriptive sensory attributes and their definitions

Attributes	Definition	Reference	Rating
Appearance			
Crust color intensity	Color intensity of the crust ranging from light brown to dark brown	Vanilla cake = 0 Chocolate brownie cake = 10	Not dark = 0 Very dark brown = 10
Crust glossiness	Light reflection on the surface	Oatmeal bread = 0 White bread = 10	Not glossy = 0 Very glossy = 10
Roughness of crust	Degree of roughness as perceived on the top surface of crumb	White bread = 0 Oatmeal bread = 10	Not rough = 0 Very rough = 10
Evenness of crust	Degree of evenness on top surface	Bread crust = 0 cookies = 10	Not even = 0 Very even = 10
Compactness	Degree of denseness of particles on top surface	White bread = 0 Cookies = 10	Not compact = 0 Very compact = 10
Sponginess	Extent of air pockets contained in sample	White bread = 0 Cookies = 10	Not spongy = 0 Very spongy = 10
Fineness of crumb	Degree of smallness of particles on surface perceived by light	White sorghum grain = 0 Mustard seeds = 10	Not fine = 0 Very fine = 10
Aroma/Smell			
Bread aroma	Aroma impression of bread and crumb after baking	Stiff porridge = 0 White fresh bread = 10	No bread aroma = 0 Intense bread aroma = 10
Malty aroma	Intensity of aroma associated with fermented yeast	Pancake = 0 Yeast bread = 10	No malty aroma = 0 Intense malty aroma = 10
Roasted meat aroma	Intensity of the aroma associated with roasted meat	Boiled meat = 0 Roasted meat = 10	No roasted meat aroma = 0 Intense roasted meat aroma = 10
Fried fish aroma	Intensity of the aroma associated with fried fish	White fresh bread = 0 Fried fish = 10	No fried fish aroma = 0 Intense fried fish aroma = 10
Cooked mushroom aroma	Intensity of the aroma associated with cooked mushroom	Cookies = 0 Cooked mushrooms = 10	No mushroom aroma = 0 Intense mushroom aroma = 10
Flavor			
Sweet flavor	Intensity of aroma associated with sugars	Distilled water without sucrose = 0 5% Sucrose in distilled water = 10	No sweet aroma = 0 Intense sweet aroma = 10
Starchy flavor	Intensity of flavor associated with cooked Irish potatoes	Cooked fresh peas = 0 Cooked Irish potatoes = 10	No starchy flavor = 0 Intense starchy flavor = 10
Fried fish flavor	Intensity of the flavor associated with fried fish	White fresh bread = 0 Fried fish = 10	No fried fish flavor = 0 Intense fried fish flavor = 10
Malty flavor	Intensity of flavor associated with fermented cereals	Pancake = 0 White fresh bread = 10	No malty flavor = 0 Intense malty flavor = 10
Texture			
Crusty texture	Noise made in the first bite of the sample between the molars	Pancake = 0 Oatmeal bread = 10	Not crusty = 0 Very crusty = 10
Chewy texture	Resilience of the sample perceived during mastication	Queen cake = 0 Oatmeal bread = 10	Not chewy = 0 Very chewy = 10
Crumby texture	Ease with which the sample is broken into smaller particles when chewing	Pancake = 0 Cookies = 10	Not crumby = 0 Very crumby = 10
Aftertaste			
Grainy residues in mouth	Degree with which mouth contains small particles after all sample has been swallowed	Pancake = 0 Roasted maize meal flour = 10	No grainy residue in mouth = 0 Intense grainy residue in mouth = 10
Malty flavor	Intensity of the flavor associated with fermented yeast	Pancake = 0 Yeast bread = 10	No malty flavor = 0 Intense malty flavor = 10
Fried fish flavor	Intensity of the flavor associated with fried fish	White fresh bread = 0 Fried fish = 10	No fried fish flavor = 0 Intense fried fish flavor = 10

Note

Supa loaf white bread (Mini Bakeries, Nairobi, Kenya) and Cookies (Paul's Bakery, Eldoret, Kenya)

#### 2.3.2 Consumer evaluation by adults

The panel was recruited through an advert to select a sample of 60 consumers. Those who responded were asked to fill a consent form informing them about the nature of samples and to ascertain their personal commitment to participate in a consumer panel to evaluate the six formulations of buns. Only those consumers who indicated their liking for buns and did not suffer any food allergies were allowed to participate. At the end, a random sample of 24 males and 36 females, aged between 20 and 35 years, was selected. Each consumer was provided with a sample of six formulations of buns, a carrot, and a glass of distilled water to cleanse their palates before and in between the tasting, all on a white tray. The consumers were asked to rate their degree of liking for appearance, aroma, flavor color, and texture on a nine‐point hedonic scale where 1 = dislike extremely, 5 = neither like nor dislike, and 9 = like extremely. The minimum value of 1 denoted not intense or not much and the maximum point of 9 denoted very intense or very much (Lawless & Heymann, 2010).

#### 2.3.3 Consumer evaluation by 8‐ to 9‐year‐old school children

**FIGURE 1:**
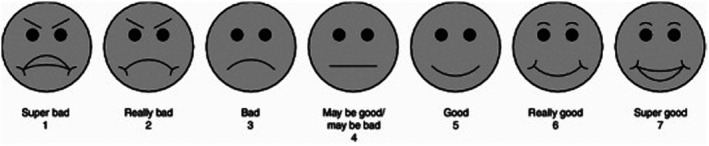
Seven‐point facial scale used by 8‐ to 9‐year‐old school children for hedonic categorization of sorghum‐wheat buns (Lawless & Heymann, 2010)

A written consent to carry out the study was sought from parents/guardians informing them about the purpose, procedures, activities, risks, and benefits of their children being involved in the study. Those children whose parents/guardians signed the consent form to allow them to participate in the study were the only ones involved. The children were also informed that they could withdraw from the study at any point if they wanted to without giving any reasons. The study was age specific so the screening selected 60 children both boys and girls aged between 8 and 9 years and did not suffer any food allergies. The study followed the policy guidelines by Schenk and Williamson (2005) on children inclusion in research. A one‐hour orientation session at 10:00 a.m. for a period of 5 days was conducted to help the children familiarize themselves on how to use a seven‐point facial scale. The sitting arrangement was designed to provide 4 groups of 15 children each. Four research assistants who were able to speak both English and Swahili were involved in handling each group. It was explained to the children that the faces on the scale are linked to super good, really good, good, maybe good or maybe bad, bad, really bad, and super bad (Figure 1).

During the orientation, two types of mangoes, one that children generally like (sweet‐ripe) and one that they generally do not like (sour‐green) labeled with three‐digits blind code were used as test samples. The children were instructed to remove the label from the mango and place it above the face corresponding to how they feel about the mango they have tasted, with the liked mango label on a super good face and the disliked mango label on a super bad face. Bottled water to cleanse the pallet before and in between tasting was also provided. Evaluation of buns was carried out in two sessions of 30 min each over a period of 3 days. During the first session, each group of children was randomly presented with six formulations of buns randomly labeled with three‐digit codes and the order of presentation also randomized. Each ¼ of the six formulations of buns were presented to each panelist on a white tray, in a glass ramekin covered with cling film, in addition to a toothpick, a serviette, carrot slices, and a plastic tumbler filled with distilled water for cleansing the palate between tasting of samples. The children tasted each bun, starting from left to right, then removed the coding labels and placed them on the scoring sheet slightly above the face that corresponded with their feelings. A similar procedure was repeated for 3 days at the same time. On each of the 3 days before the tasting began, procedures for evaluation were demonstrated to the children.

### 2.4 Data analysis

The descriptive panel mean scores for sensory attributes were determined by two‐way analysis of variance ANOVA with samples as fixed effect and panelists as random effect. Principal component analysis (PCA) of significant sensory attributes from means across panelists was performed using a correlation matrix with buns in rows and descriptors in columns. Consumer evaluation data was analyzed using a one‐way analysis of variance (ANOVA). Means for all analyses were compared using Fisher's least significant difference (LSD). Box and whisker plots were used to illustrate consumer hedonic score distributions for buns. Significance was tested at *p* ≤ .05.

## 3 RESULTS AND DISCUSSION

### 3.1 Descriptive sensory evaluation

**FIGURE 2:**
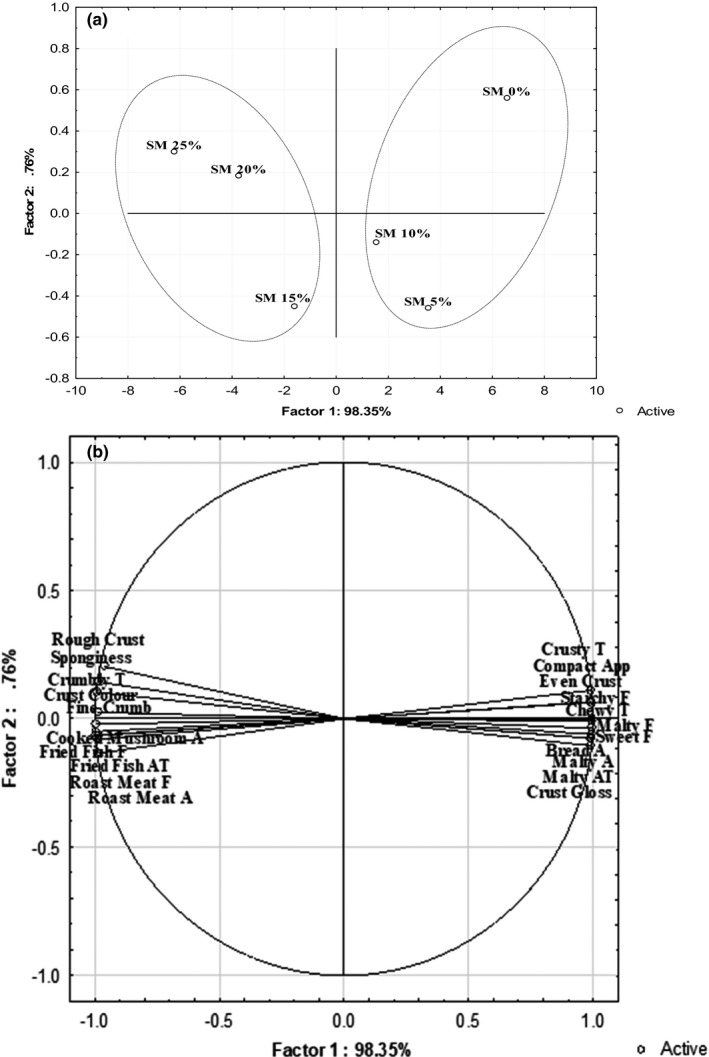
Principal component analysis (correlation matrix) of variations in SMP‐fortified buns. (a) Plot of the first two principal component scores of the buns. (b) Plot of the first two principle component loading projections of sensory attributes. Acronyms: A—Aroma, T—Texture, F—Flavor, APP—Appearance, AT—Aftertaste, SM—Snail meat

Analysis of variance (*F*‐values) for buns profile data of 23 attributes scored by the descriptive panel showed significant differences (*p* ≤ .05) between all the bun formulations (Table 2). The data was further analyzed by PCA to determine systematic variations and principal relationship among sensory attributes of buns prepared by replacing part of sorghum‐wheat flour with 5, 10, 15, 20, and 25% SMP. The first two principal components explained 99% of total variation among the six bun formulations (Figure 2a). Factor 1 explained 98% of total variation and separated buns based on proportion of SMP added with 0%–10% SMP on the right and 15%–25% SMP on the left. Factor 2 accounted for the remaining 1% of variation and separated the buns based on the physical appearance with the not‐dark bun at top right.

**TABLE 2:** Mean scores for sensory attributes of sorghum‐wheat buns as evaluated by a trained descriptive sensory panel (*n* = 8)

Attributes	Buns						
	0%	5%	10%	15%	20%	25%	*F* Value
Appearance							
Crust color intensity	3.38^a^ ± 0.65	5.08^b^ ± 0.83	5.63^c^ ± 0.49	6.71^d^ ± 0.46	7.92^e^ ± 0.41	8.92^f^ ± 0.28	319.44*
Crust glossiness	8.08^f^ ± 0.58	7.21^e^ ± 0.51	6.58^d^ ± 0.50	5.46^c^ ± 0.66	3.96^b^ ± 0.75	3.29^a^ ± 0.62	226.41*
Roughness of crust	1.46^a^ ± 0.78	1.25^a^ ± 1.11	2.13^b^ ± 0.95	2.75^c^ ± 0.61	3.29^d^ ± 0.62	3.63^d^ ± 0.49	36.14*
Evenness of crust	6.33^f^ ± 0.56	5.58^e^ ± 0.50	4.92^d^ ± 0.28	3.88^c^ ± 0.54	3.50^b^ ± 0.51	3.17^a^ ± 0.38	168.03*
Compactness	6.50^e^ ± 0.51	5.50^d^ ± 0.66	5.08^c^ ± 0.50	4.08^b^ ± 0.65	4.00^b^ ± 0.59	3.17^a^ ± 0.38	111.61*
Sponginess	3.92^a^ ± 0.41	4.50^b^ ± 0.51	5.08^c^ ± 0.50	5.63^d^ ± 0.58	6.42^e^ ± 0.78	7.25^f^ ± 0.68	105.59*
Fineness of crumb	4.17^a^ ± 0.38	4.50^b^ ± 0.51	4.92^c^ ± 0.50	5.88^d^ ± 0.34	6.58^e^ ± 0.50	7.33^f^ ± 0.56	166.25*
Aroma/Smell							
Bread aroma	8.82^f^ ± 0.58	7.63^e^ ± 0.65	6.88^d^ ± 0.61	6.33^c^ ± 0.48	5.50^b^ ± 0.51	4.83^a^ ± 0.48	136.80*
Malty aroma	7.21^f^ ± 0.83	6.38^e^ ± 0.82	5.79^d^ ± 0.83	5.29^c^ ± 0.75	4.50^b^ ± 0.59	4.00^a^ ± 0.72	58.08*
Roasted meat aroma	0.08^a^ ± 0.28	1.54^b^ ± 0.59	2.75^c^ ± 0.53	3.67^d^ ± 0.64	4.38^e^ ± 0.58	5.21^f^ ± 0.59	287.48*
Fried fish aroma	0.17^a^ ± 0.38	1.92^b^ ± 0.65	2.50^c^ ± 0.51	4.33^d^ ± 0.64	5.17^e^ ± 0.56	6.08^f^ ± 0.50	391.85*
Cooked mushroom aroma	0.67^a^ ± 0.70	1.46^b^ ± 0.59	1.96^c^ ± 0.46	2.63^d^ ± 0.58	3.04^e^ ± 0.46	3.46^f^ ± 0.59	80.33*
Flavor							
Sweet flavor	7.96^f^ ± 0.55	7.33^e^ ± 0.48	6.71^d^ ± 0.55	6.21^c^ ± 0.51	5.50^b^ ± 0.51	4.83^a^ ± 0.48	121.32*
Starchy flavor	5.75^e^ ± 0.44	5.25^d^ ± 0.44	5.08^d^ ± 0.28	4.33^c^ ± 0.48	4.08^b^ ± 0.28	3.58^a^ ± 0.50	92.03*
Roasted meat flavor	0.08^a^ ± 0.28	1.96^b^ ± 0.46	2.79^c^ ± 0.51	3.67^d^ ± 0.64	4.38^e^ ± 0.58	5.21^f^ ± 0.59	296.25*
Fried fish flavor	0.17^a^ ± 0.38	1.92^b^ ± 0.65	2.50^c^ ± 0.51	4.33^d^ ± 0.64	5.17^e^ ± 0.56	6.08^f^ ± 0.50	391.85*
Malty flavor	5.46^f^ ± 0.59	4.88^e^ ± 0.61	4.21^d^ ± 0.59	3.83^c^ ± 0.64	3.17^b^ ± 0.70	2.38^a^ ± 0.58	78.95*
Texture							
Crusty texture	6.50^e^ ± 0.51	5.50^d^ ± 0.66	5.08^c^ ± 0.50	4.08^b^ ± 0.65	4.00^b^ ± 0.59	3.17^a^ ± 0.38	111.61*
Chewy texture	5.75^e^ ± 0.44	5.25^d^ ± 0.44	5.08^d^ ± 0.28	4.33^c^ ± 0.48	4.08^b^ ± 0.28	3.58^a^ ± 0.50	92.03*
Crumby texture	3.17^a^ ± 0.38	4.00^b^ ± 0.59	4.08^b^ ± 0.65	5.08^c^ ± 0.50	5.50^d^ ± 0.66	6.50^e^ ± 0.51	111.61*
Aftertaste							
Grainy residues in mouth	3.75^f^ ± 0.44	3.25^e^ ± 0.44	2.92^d^ ± 0.28	2.33^c^ ± 0.48	2.08^b^ ± 0.50	1.83^a^ ± 0.38	71.06*
Malty flavor	4.50^f^ ± 0.59	3.96^e^ ± 0.46	3.21^d^ ± 0.59	2.83^c^ ± 0.64	2.17^b^ ± 0.70	1.42^a^ ± 0.65	82.96*
Fried fish flavor	0.00^a^ ± 0.00	1.13^b^ ± 0.54	1.54^c^ ± 0.51	2.88^d^ ± 0.74	3.50^e^ ± 0.78	4.04^f^ ± 0.75	151.92*

Note

Values are means ± standard deviations. Values followed by the same letter superscripts in the same row are not significantly different at (*p* ≤ .05) as assessed by Fischer's least significant test.

*Significantly different at (*p* < .05).

Buns incorporated at 15, 20, and 25% with SMP were associated with dark brown crust, fine crumb, sponginess, cooked mushroom and roasted meat aroma, fried fish and roasted meat flavor, crumby texture, and fried fish aftertaste (Figure 2b). Dark crust could be attributed to Maillard reaction based on the composition of buns that produced brown polymers, which contributed to coloration of buns (Serrem, Kock, & Taylor, 2011). Previously, Niaba et al. (2013) demonstrated that supplementing with defatted termite flour resulted in darker crust color. Blending with SMP also lead to a smooth dough consistency during kneading that increased pore formation in crumb after baking bringing about sponginess, fine crumb, and crumby texture of buns (Agengo et al., 2020a). The cooked mushroom, roasted meat, and fried fish flavor and aroma in buns may have been activated by thermal activities on odorants such as 1‐octen‐3‐one, 2‐acetylthiazole and tetradecanal present in processed snail meat (Lasekan et al., 2018).

Sensory attributes of crust compactness, evenness, roughness and glossiness, bread and malty aroma, starchy, sweet and malty flavor, crusty and chewy texture, and malty aftertaste were perceived in buns substituted at 0, 5, and 10% with SMP (Figure 2b). These attributes were negatively correlated with fine crumb and spongy appearance, cooked mushroom and roasted meat aroma, fried fish and roasted meat flavor, crumby texture, and fried fish aftertaste. Compactness and evenness of crust may be ascribed to reduced pore sizes due to high fiber in buns on decreasing substitution with SMP that limited expansion in gas cells (Collar et al., 2007). Roughness of crust could be associated with increased grainy particles on higher concentration of sorghum‐wheat flour in dough for making buns. Earlier, Agengo et al. (2020a) observed that buns formulated from sorghum‐wheat flour blended at high percentage with SMP had soft texture owing to low fiber content. Crust glossiness may have improved due to gelatinization of starch and dextrin formation (Altamirano‐Fortoul et al., 2012). Similarly, sweet, malty, starchy, and bread aroma and flavor in buns were possibly enhanced through the actions of kneading and fermenting dough and the thermal process of baking. A study by Ganzle et al. (2008) noted that dough mixing elicited odorants in breadcrumbs while baking developed flavor compounds in crust. Crusty and chewy texture also increased with increasing proportion of sorghum‐wheat flour in dough. These results agree with those of Agengo et al. (2020a) who demonstrated that progressive substitution of sorghum‐wheat buns with SMP reduced the hard texture characteristics associated with crusty and chewiness.

### 3.2 Consumer evaluation by adults

Adult consumer perception on appearance, aroma, flavor, and texture of buns are as presented in Table 3. Blending sorghum‐wheat buns at 20 and 25%, respectively, with SMP resulted in low consumer scores in attributes of appearance, aroma, and flavor. Low consumer rating for appearance may be attributed to the dark crust and crumb color of the buns. Other researchers have reported similar results. For instance, Mridula et al. (2007) established a lower consumer acceptability for the dark colored sorghum‐wheat composite biscuits. Similar results were also reported by Serrem, Kock, and Taylor (2011) for sorghum and bread wheat biscuits supplemented with defatted soy flour. Consistently, acceptability in flavor and aroma of sorghum‐wheat buns declined with increased quantity of SMP in dough. This is attributable to consumer unfamiliarity with SMP as an ingredient in baking. Earlier, Malik et al. (2011) assessing the nutritional and organoleptic properties of snail meat and other livestock meats determined that consumers vastly appreciated aroma and flavor of food they were familiar with such as beef and chicken, unlike snail meat.

**TABLE 3:** Mean scores for sensory attributes of snail meat powder‐fortified sorghum‐wheat buns as evaluated by adult consumers (*n* = 60)

Buns	Appearance	Aroma/Smell	Flavor	Texture
S‐WB 0% SMP	7.80^e^ ± 0.90	7.17^e^ ± 1.20	7.25^e^ ± 1.08	4.95^a^ ± 1.62
S‐WB 5% SMP	7.42^e^ ± 0.72	6.82^d^ ± 0.91	6.92^cd^ ± 0.79	5.17^a^ ± 1.38
S‐WB 10% SMP	6.92^d^ ± 1.14	6.52^d^ ± 1.26	6.68^c^ ± 0.87	5.98^b^ ± 0.97
S‐WB 15% SMP	6.37^c^ ± 1.46	6.10^c^ ± 1.07	5.63^b^ ± 1.38	6.37^b^ ± 1.01
S‐WB 20% SMP	5.82^b^ ± 1.64	5.58^b^ ± 1.49	5.20^a^ ± 1.42	6.98^c^ ± 1.03
S‐WB 25% SMP	5.35^a^ ± 1.25	5.12^a^ ± 0.88	4.97^a^ ± 1.04	7.37^d^ ± 1.07

Note

Values are means ± standard deviations. Values followed by the same letter superscripts in the same row are not significantly different at (*p* ≤ .05) as assessed by Fischer's least significant test.

Abbreviations: SMP, Snail Meat Powder; S‐WB, Sorghum‐Wheat Buns.

The control bun was rated high by adult consumers in all aspects except texture that perhaps was caused by high fiber in sorghum‐wheat composite dough, which limited expansion of gas cells leading to production of a compact and hard texture bun. These findings are in agreement with Eimam et al. (2008) who stated that bread substituted with wheat bran had tough crumby texture attributable to enhanced fiber. Texture of the control bun could also have been influenced by increased moisture retention in dough during mixing (Gomez et al., 2003). Buns substituted at between 15% and 25% with SMP scored higher in consumer rating for texture. Cakmak et al. (2013) found that replacing part of white and whole wheat bread with chicken meat and chicken meat powder on increasing concentration resulted in bread with the highest consumer ranking in texture.

**FIGURE 3:**
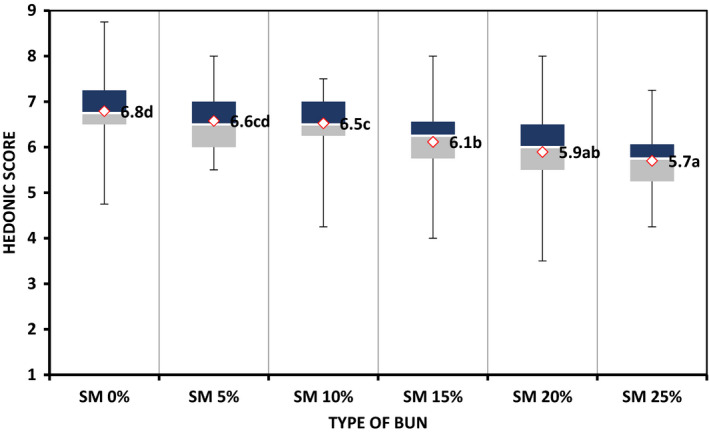
Effect of fortifying sorghum‐wheat bun with snail meat powder on total quality as evaluated by adult consumers (*n* = 60). ^abcd^ = Mean values with different letter superscripts differ significantly (*p* ≤ .05) as assessed by Fisher's least significant test. The dark shaded area is the higher percentile value above which 75% ratings fell. The light shaded area is the lower percentile area where 25% ratings fell. The median is the thin line between the two shaded areas where 50% values fell above and 50% below

Hedonic rating on total quality by adult consumers indicate that all bun formulations were preferred above average (Figure 3). However, buns substituted between 15% and 25% with SMP scored slightly lower on quality probably because of consumer unfamiliarity with intense dark color due to increased concentration of SMP in dough. Previously, Mridula et al. (2007) established low rating on total quality of soy‐fortified wheat biscuits owing to increased dark color on incorporation with sorghum flour at high proportion.

### 3.3 Consumer evaluation by 8‐ to 9‐year‐old school children

**FIGURE 4:**
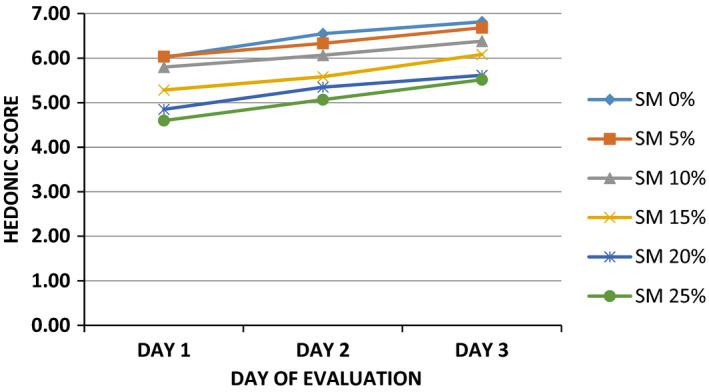
Effect of fortifying with snail meat powder on liking of sorghum‐wheat buns as evaluated by 8‐ to 9‐year‐old school children (*n* = 60) on repeated exposure

Results on the effect of fortifying with SMP on liking of sorghum‐wheat bun as evaluated by 8‐ to 9‐year‐old school children on repeated exposure are shown in Figure 4. Liking of sorghum‐wheat bun supplemented with SMP improved over time as children were repeatedly exposed to the products. This may be explained by the fact that children were not familiar with SMP as a basic ingredient in baking at initial evaluation since conventional buns are prepared from wheat and/or other cereal flour. These results are consistent with findings of Kramer et al. (2001) who observed that foods repeatedly consumed subsequently result in high rating from the minimum score when first introduced. Similar findings have been reported by Serrem, Kock, and Taylor (2011) for consumer test of sorghum and bread wheat biscuits supplemented with defatted soy flour on a repeated exposure with 8 to 9 year old schoolchildren.

Correspondingly, repeated exposure significantly did not change children's liking of the control bun over time. A possible explanation is that buns formulated from dough of staple cereal such as sorghum and wheat have sustained acceptability. Findings from previous studies have reported similar results in consumer liking. For example, Hetherington et al. (2002) revealed that consumer preference of staple cereal bread did not change over 3 weeks on repeated exposure. Therefore, food liking in children might be stimulated by other internal and external factors relating to what they like, know, and want to eat. For instance, Leon et al. (1999) established that biscuits fortified with banana and lemon were less preferred compared to those blended with strawberry, raspberry, and apricot as they were new products that children were unfamiliar with since they are not ordinarily consumed by French children.

**FIGURE 5:**
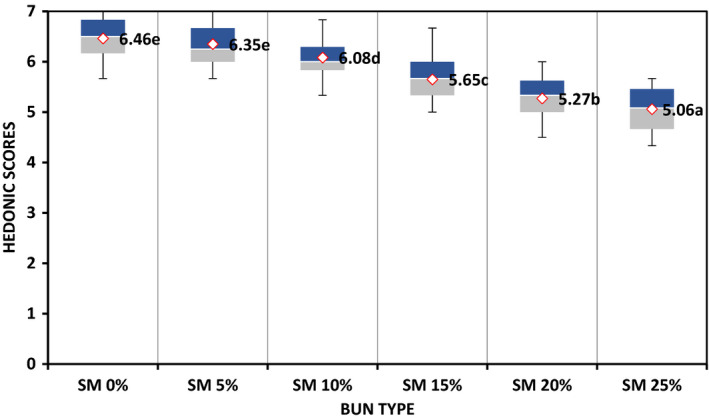
Effect of fortifying sorghum‐wheat buns with snail meat powder on agreement among consumers as evaluated by 8‐ to 9‐year‐old school children (*n* = 60). ^abcde^ = Mean values with different letter superscripts differ significantly (*p* ≤ .05) as assessed by Fisher's least significant test. The dark shaded area is the higher percentile value above which 75% ratings fell. The light shaded area is the lower percentile area where 25% ratings fell. The median is the thin line between the two shaded areas where 50% values fell above and 50% below

Figure 5 shows findings on effect of fortifying buns with SMP on consumer agreement as determined by 8‐ to 9‐year‐old schoolchildren. The shorter distribution along the bar line of graph in this study indicates agreement among children on score for buns and that results were consistent. These results agree with findings of Leon et al. (1999) who established that children of 8‐ to 9‐year‐old were more consistent in describing their liking for food products. Positive hedonic rating of buns fortified with SMP indicate that the children were not bored with the samples.

## 4 CONCLUSION

Compositing sorghum‐wheat with SMP imparts positive consumer attributes of fine crumb, spongy, and crumby texture, while reducing proportions of SMP in buns results in compact and rough crust and chewy texture. Buns formulated from sorghum‐wheat flour blended with SMP have reasonably high acceptability and 8‐ to 9‐year‐old school children could sustain their acceptability over time on repeated exposure as supplementary‐rich sources of protein for alleviating the menace of protein energy malnutrition in sub‐Saharan Africa.

## CONFLICT OF INTEREST

The authors declare no conflict of interest.

## ETHICAL APPROVAL

The study approval was granted by the Jomo Kenyatta University of Agriculture and Technology, Juja Kenya (REF: JKU/2/11/AG422‐4603/2015).

## INFORMED CONSENT

Permission to conduct consumer studies by children was granted by parents/guardians, and those children who voluntarily accepted and whose guardians signed the consent form, which informed them of the nature of samples and activities involved in the study, were included. An informed and written consent was sought from participants before the study commenced.

## Data Availability

Data Availability statement for the data used in your manuscript.
All data created during this research is openly available at the Jomo Kenyatta University of Agriculture and Technology, Juja ‐ Kenya, school of Food and Nutritional Sciences Research Data Archive. All data supporting this study are provided in full in the results section of this paper All the supporting data is also openly available from fagengo@yahoo.co.uk
